# 3,5-Dicarboxyanilinium nitrate dihydrate

**DOI:** 10.1107/S1600536810005362

**Published:** 2010-02-20

**Authors:** Wen-Xian Liang, Yun-Ti Zhu

**Affiliations:** a Ordered Matter Science Research Center, College of Chemistry and Chemical Engineering, Southeast University, Nanjing 210096, People’s Republic of China

## Abstract

In the crystal of the title compound, C_8_H_8_NO_4_^+^·NO_3_^−^·2H_2_O, the 5-ammonio­isophthalic acid cations, the nitrate anions and the water mol­ecules are linked by N—H⋯O, O—H⋯O and C—H ⋯O hydrogen bonds into a three-dimensional network. The structure is further stabilized by aromatic π–π stacking inter­actions, with centroid–centroid separations of 3.827 (2) Å.

## Related literature

For the crystal structure of 5-amino­isophthalic acid hemihydrate, see: Dobson *et al.* (1998 ▶). For the use of 5-amino­isophthalic acid as a ligand, see: Liao *et al.* (2004 ▶).
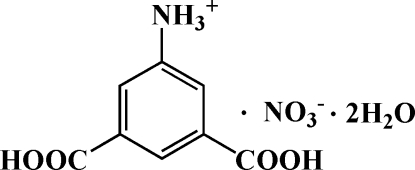


## Experimental

### 

#### Crystal data

C_8_H_8_NO_4_^+^·NO_3_^−^·2H_2_O
*M* *_r_* = 280.20
Monoclinic, 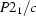

*a* = 8.3436 (17) Å
*b* = 8.6234 (17) Å
*c* = 16.862 (3) Å
β = 97.31 (3)°
*V* = 1203.4 (4) Å^3^
*Z* = 4
Mo *K*α radiation
μ = 0.14 mm^−1^
*T* = 293 K
0.35 × 0.25 × 0.10 mm

#### Data collection

Rigaku SCXmini diffractometer
Absorption correction: multi-scan (*CrystalClear*; Rigaku, 2005 ▶) *T*_min_ = 0.960, *T*_max_ = 0.986
12169 measured reflections
2753 independent reflections
1905 reflections with *I* > 2σ(*I*)
*R*_int_ = 0.055

#### Refinement

*R*[*F* ^2^ > 2σ(*F* ^2^)] = 0.054
*wR*(*F* ^2^) = 0.129
*S* = 1.07
2753 reflections
190 parameters
?
Δρ_max_ = 0.28 e Å^−3^
Δρ_min_ = −0.24 e Å^−3^

### 

Data collection: *CrystalClear* (Rigaku 2005 ▶); cell refinement: *CrystalClear*; data reduction: *CrystalClear*; program(s) used to solve structure: *SHELXS97* (Sheldrick, 2008 ▶); program(s) used to refine structure: *SHELXL97* (Sheldrick, 2008 ▶); molecular graphics: *SHELXTL* (Sheldrick, 2008 ▶); software used to prepare material for publication: *PRPKAPPA* (Ferguson, 1999 ▶).

## Supplementary Material

Crystal structure: contains datablocks I, global. DOI: 10.1107/S1600536810005362/rz2414sup1.cif

Structure factors: contains datablocks I. DOI: 10.1107/S1600536810005362/rz2414Isup2.hkl

Additional supplementary materials:  crystallographic information; 3D view; checkCIF report

## Figures and Tables

**Table 1:** Hydrogen-bond geometry (Å, °)

*D*—H⋯*A*	*D*—H	H⋯*A*	*D*⋯*A*	*D*—H⋯*A*
C2—H2⋯O6^i^	0.93	2.58	3.324 (3)	138
O9—H9*A*⋯O7^ii^	0.88 (4)	2.39 (4)	3.048 (3)	133 (3)
O9—H9*A*⋯O5^ii^	0.88 (4)	1.94 (4)	2.801 (3)	168 (4)
O8—H8*B*⋯O3^iii^	0.85 (5)	2.29 (5)	3.057 (3)	149 (4)
O8—H8*A*⋯O2^iv^	0.97 (4)	2.00 (4)	2.882 (3)	151 (3)
O4—H4⋯O3^v^	0.82	1.84	2.652 (2)	169
N1—H1*C*⋯O9^vi^	0.89	1.98	2.839 (3)	163
N1—H1*B*⋯O6^vii^	0.89	2.00	2.859 (3)	161
N1—H1*B*⋯O5^vii^	0.89	2.55	3.134 (3)	124
O9—H9*B*⋯O8	0.84 (5)	1.96 (5)	2.796 (3)	171 (5)
N1—H1*A*⋯O7^i^	0.89	2.46	2.933 (3)	114
N1—H1*A*⋯O6^i^	0.89	2.09	2.963 (3)	165
O1—H1⋯O9^viii^	0.82	1.80	2.611 (3)	169

Symmetry codes: (i) 
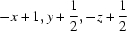
; (ii) 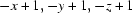; (iii) 
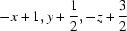
; (iv) 
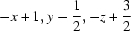
; (v) 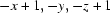; (vi) 
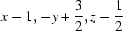
; (vii) 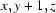; (viii) 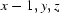.

**Fig. 1.**
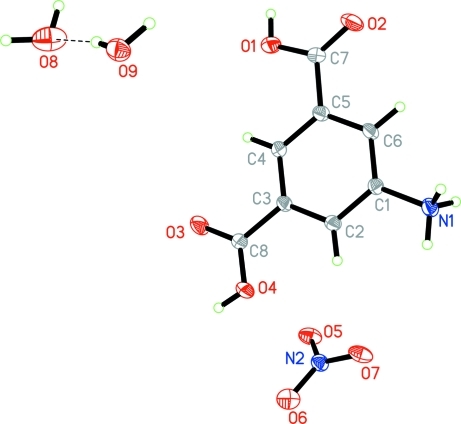
The asymmetric unit of the title compound, with displacement ellipsoids drawn at the 30% probability level. Intermolecular hydrogen bonds are shown as dashed lines.

**Fig. 2.**
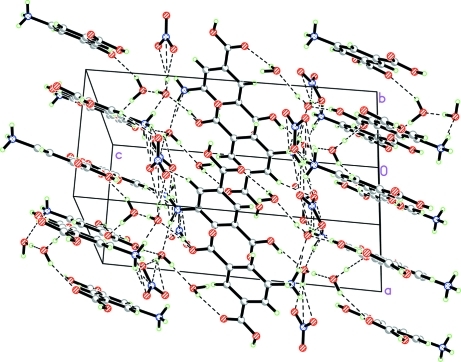
Packing diagram of the title compound. Hydrogen bonds are shown as dashed lines.

## supplementary crystallographic information

### Comment

5-Aminobenzene-1,3-dioic acid (5-aminoisophthalic acid) is an important molecule due to its amphoteric property. The report on 5-aminobenzene-1,3-dioic acid hemihydrate (Dobson *et al.* 1998) is one of a series on hydrogen bonding in aminosubstituted carboxylic acids, and follows reports on a novel tetragonal phase of aminobutyric acid, on 8-aminocaprylic acid and on 3-aminoisobutyric acid monohydrate. In addition, 5-aminobenzene-1,3-dioic acid is an attractive ligand for use in the generation of polar coordination polymers (Liao *et al.*, 2004).

The asymmetric unit of the title compound comprises two water molecules, a 5-ammonioisophthalic acid cation and one nitrate anion (Fig. 1). The crystal packing is stabilized by hydrogen bonds of N—H···O, O—H···O, C—H···O (Table 1) connecting neighbouring water molecules, cations and anions into a three-dimensional network (Fig. 2). The structure is further stabiized by aromatic π···π stacking interactions, with centroid-to-centroid separations of 3.827 (2) Å.

### Experimental

5-Aminoisophthalic acid (1.81 g, 10 mmol) was dissolved in water (5 ml), ethanol (20 ml) and nitric acid (0.57 g, 10 mmol) and the solution was filtered. After slowly evaporating over a period of 3 d, colourless prismatic crystals of the title compound suitable for X-ray diffraction analysis were isolated.

### Refinement

All the H atoms were calculated geometrically and were allowed to ride on their parent atomsd, with C—H = 0.93–0.97 Å, N—H = 0.89 Å, and with U_iso_(H) = 1.2Ueq(C) and 1.5U_eq_(N).

### Crystal data

**Table:**

C_8_H_8_NO_4_^+^·NO_3_^−^·2H_2_O	*F*(000) = 584
*M**_r_* = 280.20	*D*_x_ = 1.547 Mg m^−^^3^
Monoclinic, *P*2_1_/*c*	Mo *K*α radiation, λ = 0.71073 Å
Hall symbol: -P 2ybc	Cell parameters from 1753 reflections
*a* = 8.3436 (17) Å	θ = 3.1–27.5°
*b* = 8.6234 (17) Å	µ = 0.14 mm^−^^1^
*c* = 16.862 (3) Å	*T* = 293 K
β = 97.31 (3)°	Prism, colourless
*V* = 1203.4 (4) Å^3^	0.35 × 0.25 × 0.10 mm
*Z* = 4	

### Data collection

**Table:**

Rigaku SCXmini diffractometer	2753 independent reflections
Radiation source: fine-focus sealed tube	1905 reflections with *I* > 2σ(*I*)
graphite	*R*_int_ = 0.055
Detector resolution: 13.6612 pixels mm^-1^	θ_max_ = 27.5°, θ_min_ = 3.2°
CCD profile fitting scans	*h* = −10→10
Absorption correction: multi-scan (*CrystalClear*; Rigaku, 2005)	*k* = −11→11
*T*_min_ = 0.960, *T*_max_ = 0.986	*l* = −21→21
12169 measured reflections	

### Refinement

**Table:**

Refinement on *F*^2^	Secondary atom site location: difference Fourier map
Least-squares matrix: full	Hydrogen site location: inferred from neighbouring sites
*R*[*F*^2^ > 2σ(*F*^2^)] = 0.054	*w* = 1/[σ^2^(*F*_o_^2^) + (0.0423*P*)^2^ + 0.7735*P*] where *P* = (*F*_o_^2^ + 2*F*_c_^2^)/3
*wR*(*F*^2^) = 0.129	(Δ/σ)_max_ < 0.001
*S* = 1.07	Δρ_max_ = 0.28 e Å^−^^3^
2753 reflections	Δρ_min_ = −0.24 e Å^−^^3^
190 parameters	Extinction correction: *SHELXL97* (Sheldrick, 2008)
0 restraints	Extinction coefficient: 0.0014 (1)
Primary atom site location: structure-invariant direct methods	

### Special details

**Table:**

Geometry. All esds (except the esd in the dihedral angle between two l.s. planes) are estimated using the full covariance matrix. The cell esds are taken into account individually in the estimation of esds in distances, angles and torsion angles; correlations between esds in cell parameters are only used when they are defined by crystal symmetry. An approximate (isotropic) treatment of cell esds is used for estimating esds involving l.s. planes.
Refinement. Refinement of *F*^2^ against ALL reflections. The weighted *R*-factor wR and goodness of fit *S* are based on *F*^2^, conventional *R*-factors *R* are based on *F*, with *F* set to zero for negative *F*^2^. The threshold expression of *F*^2^ > σ(*F*^2^) is used only for calculating *R*-factors(gt) etc. and is not relevant to the choice of reflections for refinement. *R*-factors based on *F*^2^ are statistically about twice as large as those based on *F*, and *R*- factors based on ALL data will be even larger.

### Fractional atomic coordinates and isotropic or equivalent isotropic displacement parameters (Å^2^)

**Table:**

	*x*	*y*	*z*	*U*_iso_*/*U*_eq_	
O4	0.4952 (2)	0.14675 (19)	0.42859 (10)	0.0442 (5)	
H4	0.5275	0.0603	0.4432	0.066*	
N1	0.3108 (2)	0.6452 (2)	0.29415 (11)	0.0328 (5)	
H1A	0.3651	0.5813	0.2658	0.049*	
H1B	0.3632	0.7350	0.3010	0.049*	
H1C	0.2126	0.6614	0.2682	0.049*	
C2	0.3575 (3)	0.4279 (2)	0.38793 (13)	0.0280 (5)	
H2	0.4070	0.3744	0.3498	0.034*	
O1	0.1136 (3)	0.6025 (2)	0.62566 (11)	0.0545 (6)	
H1	0.0669	0.6559	0.6558	0.082*	
O3	0.3885 (3)	0.1368 (2)	0.54276 (11)	0.0513 (5)	
C4	0.2667 (3)	0.4405 (2)	0.51780 (13)	0.0296 (5)	
H4A	0.2556	0.3946	0.5667	0.036*	
C1	0.2969 (3)	0.5756 (2)	0.37216 (13)	0.0264 (5)	
C5	0.2069 (3)	0.5891 (3)	0.50071 (14)	0.0299 (5)	
C6	0.2225 (3)	0.6570 (2)	0.42699 (13)	0.0300 (5)	
H6	0.1830	0.7563	0.4152	0.036*	
O2	0.0772 (3)	0.8101 (2)	0.54705 (12)	0.0605 (6)	
C3	0.3432 (3)	0.3610 (2)	0.46132 (13)	0.0275 (5)	
C7	0.1260 (3)	0.6799 (3)	0.55994 (14)	0.0356 (6)	
O5	0.2218 (2)	−0.0029 (2)	0.30373 (12)	0.0461 (5)	
O6	0.4607 (2)	−0.06093 (19)	0.27628 (11)	0.0441 (5)	
O7	0.3495 (2)	0.15470 (19)	0.23390 (12)	0.0460 (5)	
N2	0.3433 (2)	0.0312 (2)	0.27105 (12)	0.0333 (5)	
C8	0.4106 (3)	0.2034 (3)	0.47971 (13)	0.0304 (5)	
O9	0.9920 (2)	0.7569 (2)	0.73715 (12)	0.0422 (5)	
O8	0.8512 (3)	0.5594 (3)	0.84014 (16)	0.0667 (7)	
H9A	0.916 (5)	0.828 (5)	0.729 (2)	0.091 (13)*	
H8A	0.904 (4)	0.500 (5)	0.885 (2)	0.089 (13)*	
H8B	0.781 (6)	0.612 (5)	0.861 (3)	0.111 (16)*	
H9B	0.949 (6)	0.706 (6)	0.771 (3)	0.14 (2)*	

### Atomic displacement parameters (Å^2^)

**Table:**

	*U*^11^	*U*^22^	*U*^33^	*U*^12^	*U*^13^	*U*^23^
O4	0.0653 (12)	0.0295 (9)	0.0394 (10)	0.0196 (9)	0.0133 (9)	0.0042 (7)
N1	0.0348 (11)	0.0289 (10)	0.0359 (11)	0.0043 (8)	0.0092 (9)	0.0069 (8)
C2	0.0321 (12)	0.0225 (11)	0.0299 (12)	0.0021 (9)	0.0057 (10)	−0.0010 (9)
O1	0.0824 (15)	0.0496 (11)	0.0354 (10)	0.0243 (10)	0.0225 (10)	0.0014 (9)
O3	0.0760 (14)	0.0357 (10)	0.0470 (11)	0.0214 (10)	0.0261 (10)	0.0177 (8)
C4	0.0365 (13)	0.0251 (11)	0.0269 (12)	0.0028 (9)	0.0025 (10)	0.0010 (9)
C1	0.0283 (11)	0.0236 (11)	0.0275 (11)	−0.0002 (9)	0.0038 (9)	0.0026 (9)
C5	0.0323 (12)	0.0258 (11)	0.0313 (12)	0.0017 (9)	0.0029 (10)	−0.0025 (9)
C6	0.0345 (12)	0.0204 (10)	0.0348 (12)	0.0054 (9)	0.0027 (10)	0.0003 (9)
O2	0.0920 (16)	0.0371 (11)	0.0573 (13)	0.0270 (11)	0.0282 (12)	0.0011 (9)
C3	0.0317 (12)	0.0209 (10)	0.0293 (12)	0.0016 (9)	0.0021 (9)	0.0008 (9)
C7	0.0421 (14)	0.0308 (13)	0.0343 (13)	0.0059 (11)	0.0063 (11)	−0.0055 (10)
O5	0.0385 (10)	0.0427 (10)	0.0604 (12)	−0.0026 (8)	0.0199 (9)	0.0066 (9)
O6	0.0436 (10)	0.0346 (9)	0.0562 (12)	0.0130 (8)	0.0149 (9)	0.0030 (8)
O7	0.0483 (11)	0.0269 (9)	0.0643 (12)	−0.0019 (8)	0.0128 (9)	0.0126 (8)
N2	0.0363 (11)	0.0263 (10)	0.0377 (11)	−0.0009 (9)	0.0063 (9)	−0.0016 (8)
C8	0.0379 (13)	0.0246 (11)	0.0290 (12)	0.0042 (10)	0.0049 (10)	−0.0001 (9)
O9	0.0356 (10)	0.0465 (11)	0.0460 (11)	0.0073 (9)	0.0106 (9)	−0.0011 (9)
O8	0.0748 (16)	0.0582 (14)	0.0742 (17)	0.0179 (12)	0.0371 (14)	0.0138 (12)

### Geometric parameters (Å, °)

**Table:**

O4—C8	1.279 (3)	C4—H4A	0.9300
O4—H4	0.8200	C1—C6	1.371 (3)
N1—C1	1.464 (3)	C5—C6	1.395 (3)
N1—H1A	0.8900	C5—C7	1.496 (3)
N1—H1B	0.8900	C6—H6	0.9300
N1—H1C	0.8900	O2—C7	1.205 (3)
C2—C1	1.383 (3)	C3—C8	1.488 (3)
C2—C3	1.384 (3)	O5—N2	1.249 (2)
C2—H2	0.9300	O6—N2	1.256 (2)
O1—C7	1.309 (3)	O7—N2	1.240 (2)
O1—H1	0.8200	O9—H9A	0.88 (4)
O3—C8	1.243 (3)	O9—H9B	0.84 (5)
C4—C5	1.392 (3)	O8—H8A	0.97 (4)
C4—C3	1.393 (3)	O8—H8B	0.85 (5)
			
C8—O4—H4	109.5	C6—C5—C7	118.5 (2)
C1—N1—H1A	109.5	C1—C6—C5	119.2 (2)
C1—N1—H1B	109.5	C1—C6—H6	120.4
H1A—N1—H1B	109.5	C5—C6—H6	120.4
C1—N1—H1C	109.5	C2—C3—C4	120.3 (2)
H1A—N1—H1C	109.5	C2—C3—C8	119.59 (19)
H1B—N1—H1C	109.5	C4—C3—C8	120.1 (2)
C1—C2—C3	119.0 (2)	O2—C7—O1	124.5 (2)
C1—C2—H2	120.5	O2—C7—C5	122.6 (2)
C3—C2—H2	120.5	O1—C7—C5	112.9 (2)
C7—O1—H1	109.5	O7—N2—O5	120.9 (2)
C5—C4—C3	119.7 (2)	O7—N2—O6	119.8 (2)
C5—C4—H4A	120.2	O5—N2—O6	119.32 (19)
C3—C4—H4A	120.2	O3—C8—O4	123.7 (2)
C6—C1—C2	121.8 (2)	O3—C8—C3	120.5 (2)
C6—C1—N1	119.39 (19)	O4—C8—C3	115.8 (2)
C2—C1—N1	118.78 (19)	H9A—O9—H9B	96 (4)
C4—C5—C6	119.9 (2)	H8A—O8—H8B	103 (4)
C4—C5—C7	121.6 (2)		

### Hydrogen-bond geometry (Å, °)

**Table:**

*D*—H···*A*	*D*—H	H···*A*	*D*···*A*	*D*—H···*A*
C2—H2···O6^i^	0.93	2.58	3.324 (3)	138
O9—H9A···O7^ii^	0.88 (4)	2.39 (4)	3.048 (3)	133 (3)
O9—H9A···O5^ii^	0.88 (4)	1.94 (4)	2.801 (3)	168 (4)
O8—H8B···O3^iii^	0.85 (5)	2.29 (5)	3.057 (3)	149 (4)
O8—H8A···O2^iv^	0.97 (4)	2.00 (4)	2.882 (3)	151 (3)
O4—H4···O3^v^	0.82	1.84	2.652 (2)	169
N1—H1C···O9^vi^	0.89	1.98	2.839 (3)	163
N1—H1B···O6^vii^	0.89	2.00	2.859 (3)	161
N1—H1B···O5^vii^	0.89	2.55	3.134 (3)	124
O9—H9B···O8	0.84 (5)	1.96 (5)	2.796 (3)	171 (5)
N1—H1A···O7^i^	0.89	2.46	2.933 (3)	114
N1—H1A···O6^i^	0.89	2.09	2.963 (3)	165
O1—H1···O9^viii^	0.82	1.80	2.611 (3)	169

Symmetry codes: (i) −*x*+1, *y*+1/2, −*z*+1/2; (ii) −*x*+1, −*y*+1, −*z*+1; (iii) −*x*+1, *y*+1/2, −*z*+3/2; (iv) −*x*+1, *y*−1/2, −*z*+3/2; (v) −*x*+1, −*y*, −*z*+1; (vi) *x*−1, −*y*+3/2, *z*−1/2; (vii) *x*, *y*+1, *z*; (viii) *x*−1, *y*, *z*.

## References

[bb1] Dobson, A. J. & Gerkin, R. E. (1998). *Acta Cryst.* C**54**, 1503–1505. 10.1107/s0108270198005915 9807799

[bb2] Ferguson, G. (1999). *PRPKAPPA* University of Guelph, Canada.

[bb3] Liao, Q.-X., Li, Z.-J., Zhang, J., Kang, Y., Dai, Y.-M. & Yao, Y.-G. (2004). *Acta Cryst.* C**60**, m509–m511. 10.1107/S0108270104020426 15467124

[bb4] Rigaku (2005). *CrystalClear.* Rigaku Corporation, Tokyo, Japan.

[bb5] Sheldrick, G. M. (2008). *Acta Cryst.* A**64**, 112–122. 10.1107/S0108767307043930 18156677

